# Effect of Brachytherapy vs. External Beam Radiotherapy on Sexual Function in Patients With Clinically Localized Prostate Cancer: A Meta-Analysis

**DOI:** 10.3389/fcell.2021.792597

**Published:** 2022-01-19

**Authors:** Xiaodu Xie, Yuanfeng Zhang, Chengguo Ge, Peihe Liang

**Affiliations:** Department of Urology, The Second Affiliated Hospital of Chongqing Medical University, Chongqing, China

**Keywords:** localized prostate cancer, brachytherapy, external beam radiotherapy, sexual function, meta-analysis

## Abstract

**Purpose:** The aim of this study was to compare the effect of brachytherapy (BT) versus external beam radiotherapy (EBRT) on sexual function in patients with localized prostate cancer (PCa).

**Methods:** Data were retrieved from the PubMed, Cochrane Library, Embase, China National Knowledge Infrastructure (CNKI), and Wanfang Database until March 4, 2021. Analysis was performed by using RevMan 5.4.1. The main clinical outcomes were the Prostate Cancer Symptom Indices (PCSI) scale and the Expanded Prostate Cancer Index Composite (EPIC) scale scores for sexual function. A meta-analysis was performed to calculate standardized mean differences (SMDs) and their 95% CI. This study has undergone PROSPERO registration (No. CDR42021245438).

**Results:** Among the 962 studies retrieved, eight prospective cohort studies met the inclusion criteria, covering a total of 2,340 patients, including 1,138 treated with BT alone and 1,202 treated with EBRT alone. The results demonstrated that BT was to some extent advantageous over EBRT in overall sexual function scores in patients with localized PCa during the immediate post-treatment period (SMD = −0.09, 95% CI: −0.18 to −0.01, *p* = 0.03), but this difference was not detectable at 3 months (SMD = −0.07, 95% CI: −0.18–0.05, and *p* = 0.25), 12 months (SMD = −0.01, 95% CI: −0.21–0.20, and *p* = 0.96), and 24 months (SMD = −0.09, 95% CI: −0.20–0.01, and *p* = 0.09) after treatment.

**Conclusion:** Our analysis showed that BT showed a short-term advantage over EBRT in terms of sexual function in patients with localized PCa, but this difference diminished over time, though the conclusion needs to be further verified by a longer-term follow-up study.

## 1 Introduction

Prostate cancer (PCa) is the most common malignancy of the male genitourinary system. According to the International Agency for Research on Cancer (IARC) GLOBOCAN database in 2020, the global incidence of PCa is second only to lung cancer among male malignancies (Sung et al., 2021). The widespread use of serum prostate-specific antigen (PSA) screening tests has greatly increased the detection rate of localized PCa (Hayes and Barry, 2014). The treatment for PCa includes radical prostatectomy, radiotherapy, endocrine therapy, and chemotherapy, among which radical prostatectomy and radiotherapy are curative treatments mainly for localized PCa (Wallis et al., 2018). The basic principle of radiotherapy is using ionizing radiation to kill tumor cells. The applicable population is mainly PCa patients with lesions confined to the pelvis (clinically T_1–4_N_0–1_M_0_) and patients who are in a reasonably good physiological state and can tolerate possible serious comorbidities. In addition, it can also be used for remedial treatment of local recurrence after radical surgery. Radiotherapy for PCa mainly consists of external beam radiotherapy (EBRT) and brachytherapy (BT). Many studies have been reported to compare the efficacy of these two treatments. For instance, a meta-analysis reported that BT alone was superior to EBRT alone in low-risk patients with localized PCa in terms of 5-year biochemical progression-free survival (PFS), overall survival (OS), and the incidence of gastrointestinal (GI) toxicity (Li et al., 2018), while other studies argued that the 5-year PFS of BT was superior to that of EBRT only in intermediate and high-risk patients, and with no significant difference in OS or the incidence of GI toxicity (Kee et al., 2018).

The ultimate goal of tumor treatment is no longer simply to remove the tumor or prolong the survival of the patient. More importantly, maintenance of a high quality of life has become one of the basic requirements of cancer treatment. One of the most important consequences of PCa treatment is the loss of sexual function. There has been a consensus that radical surgery with preservation of the neurovascular bundle (NVB) as tumor conditions permit is beneficial for the recovery of postoperative sexual function and urinary control. However, existing evidence related to the effects of different radiotherapy methods on sexual function is scarce, and the conclusions are inconsistent (Hunt et al., 2021). The aim of this meta-analysis is to compare the effects of BT and EBRT on sexual function by retrieving the relevant literature and extracting data on sexual function scores from the Expanded Prostate Cancer Index Composite (EPIC) and Prostate Cancer Symptom Indices (PCSI) scales, aiming to provide an evidence-based basis for the selection of treatments for early-stage localized PCa in clinical work.

## 2 Materials and Methods

### 2.1 Criteria for Study Selection

According to the PICOS, we developed inclusion criteria: 1) participants (P)—all the patients were diagnosed with localized PCa without infiltration or invasion of the prostate outside the envelope or adjacent organs, without lymph node metastasis, and with a follow-up period ≥3 months after radiotherapy. 2) Interventions (I) and comparisons (C): comparing the efficacy on sexual function of BT versus EBRT. 3) Outcomes (O): the indicators were the scores of the PCSI scale and the EPIC scale regarding sexual function. 4) Study design (S): prospective and retrospective studies (including cohort studies and case–control studies).

We excluded the following articles: 1) the literature without relevant indicators; 2) duplicate publications; 3) reviews; 4) animal experiments; and 5) conference abstracts.

### 2.2 Search Strategy

We identified relevant studies by searching PubMed, Cochrane Library, Embase, China National Knowledge Infrastructure (CNKI), and Wanfang database up to March 4, 2021. The search was performed using MeSH terms, such as “Prostatic Neoplasms,” “Brachytherapy,” “Radiotherapy,” and “Erectile Dysfunction.” In addition, we scanned through the reference lists of included studies to find additional pertinent articles. We also contacted the corresponding author to acquire information if the research results were incomplete or could not be found.

### 2.3 Data Extraction

Two authors independently screened the titles and abstracts of retrieved articles and then reviewed the full texts according to the inclusion and exclusion criteria. Then, the two authors extracted the data available in the included studies to fill out the well-designed form and check with each other. Any disagreement between the two authors was reviewed and resolved through a third author. For articles that only provided data such as the mean, sample size, and CI, RevMan calculator was used to convert them and calculate the required variables for the statistics. For articles that only provided X–Y scatter plots, WebPlotDigitizer was used to extract the data.

### 2.4 Quality Assessment

The Newcastle–Ottawa Scale (NOS) was used to evaluate the quality of the included studies from the following aspects, with the maximum score of 9 points: 1) representativeness of the exposed cohort; 2) selection of the nonexposed cohort; 3) ascertainment of exposure; 4) demonstration that the outcome of interest was not present at the time of initiating the study; 5) comparability of cohorts on the basis of the design or analysis; 6) assessment of the outcome; 7) the follow-up period long enough for the outcome to occur; and 8) adequacy of follow-up of cohorts. The studies with scores ≥6 were considered as high quality, and those with scores <6 were considered as low quality.

The risk of bias was assessed in four domains—selection bias, loss to follow-up bias, information bias, and confounding bias—based on which the risk of bias was classified as “low risk,” “unknown risk,” and “high risk.”

### 2.5 Data Synthesis and Analysis

The meta-analysis was performed using the RevMan 5.4.1 software provided by the Cochrane Collaboration. Continuous data are presented as standardized mean difference (SMD) as effect size, with 95% CI calculated. Heterogeneity was evaluated by using the chi-square test and I^2^ test. If there was no significant statistical difference (*p* > 0.05, I^2^ < 50%), the fixed-effects model was used for analysis, and otherwise, the random-effects model was used. Heterogeneity was dealt with through subgroup analysis or sensitivity analysis. The means and SDs of the baseline were assumed to be X1 and S1, and the means and SD of the endpoint were X2 and S2. Then, we input “mean = X2 − X1” and “SD = $\sqrt{{\mathrm{S1}}^{2}+{\mathrm{S2}}^{2}-2*R*\mathrm{S1}*\mathrm{S2}}$ (R = 0.5)” into the RevMan to make forest plots, which made the effect of the intervention mainly through the change in the amount of effect before and after the intervention, further eliminating the effect of the baseline.

## 3 Results

### 3.1 Study Selection

**FIGURE 1:**
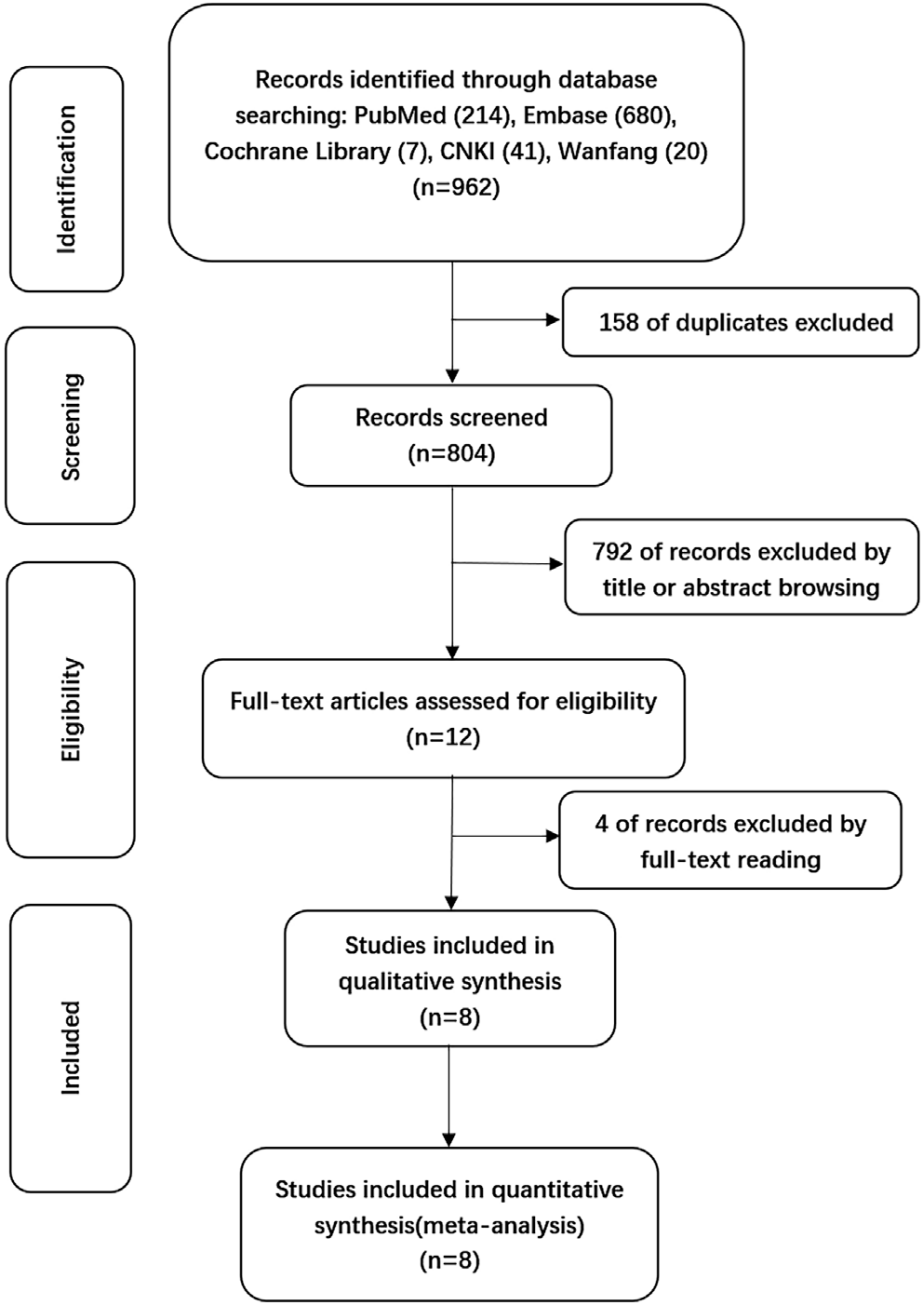
Flow diagram of the selection process.

A total of 962 citations were obtained through electronic databases, and 158 duplicates were eliminated by using EndNoteX9 software. After the titles and abstracts of the remaining 804 articles were screened, 792 articles we excluded due to irrelevancy. The full texts of the remaining 12 articles were reviewed, and finally, 8 articles were included for formal analysis according to the inclusion criteria, as detailed in Figure 1.

### 3.2 Characteristics and Quality Assessment of the Included Studies

All the eight included studies were observational cohort studies, containing 2,340 patients who met the predefined inclusion criteria. All included studies involved the comparison of the effects of BT versus EBRT on sexual function, with 1,202 patients receiving EBRT and 1,138 patients receiving BT. The NOS scores of all the 8 articles were ≥6, with a mean score of 7.0. The basic characteristics are shown in Table 1.

**TABLE 1:** Characteristics of the included studies for analysis.

Study year	Study design	NOS score	Scale	EBRT (sample size)	BT (sample size)	Total follow-up period (months)
Jia Bin et al. (2018)	Prospective cohort study	6	PCSI	63	46	**24**
Chen et al. (2017)	Prospective cohort study	8	PCSI	249	109	**24**
Mullins et al. (2019)	Prospective cohort study	7	PCSI	188	122	**24**
Ferrer et al. (2008)	Prospective cohort study	8	EPIC	205	275	**24**
Guedea et al. (2009)	Prospective cohort study	7	EPIC	134	56	**24**
Pardo et al. (2010)	Prospective cohort study	7	EPIC	127	185	**36**
Geerdink et al. (2013)	Prospective cohort study	6	EPIC	42	28	**12**
Guedea et al. (2013)	Prospective cohort study	7	EPIC	194	317	**60**

Note. NOS, Newcastle–Ottawa Scale; EBRT, external beam radiotherapy; BT, brachytherapy; PCSI, Prostate Cancer Symptom Indices; EPIC, Expanded Prostate Cancer Index Composite.

### 3.3 Risk of Bias Assessment

**FIGURE 2:**
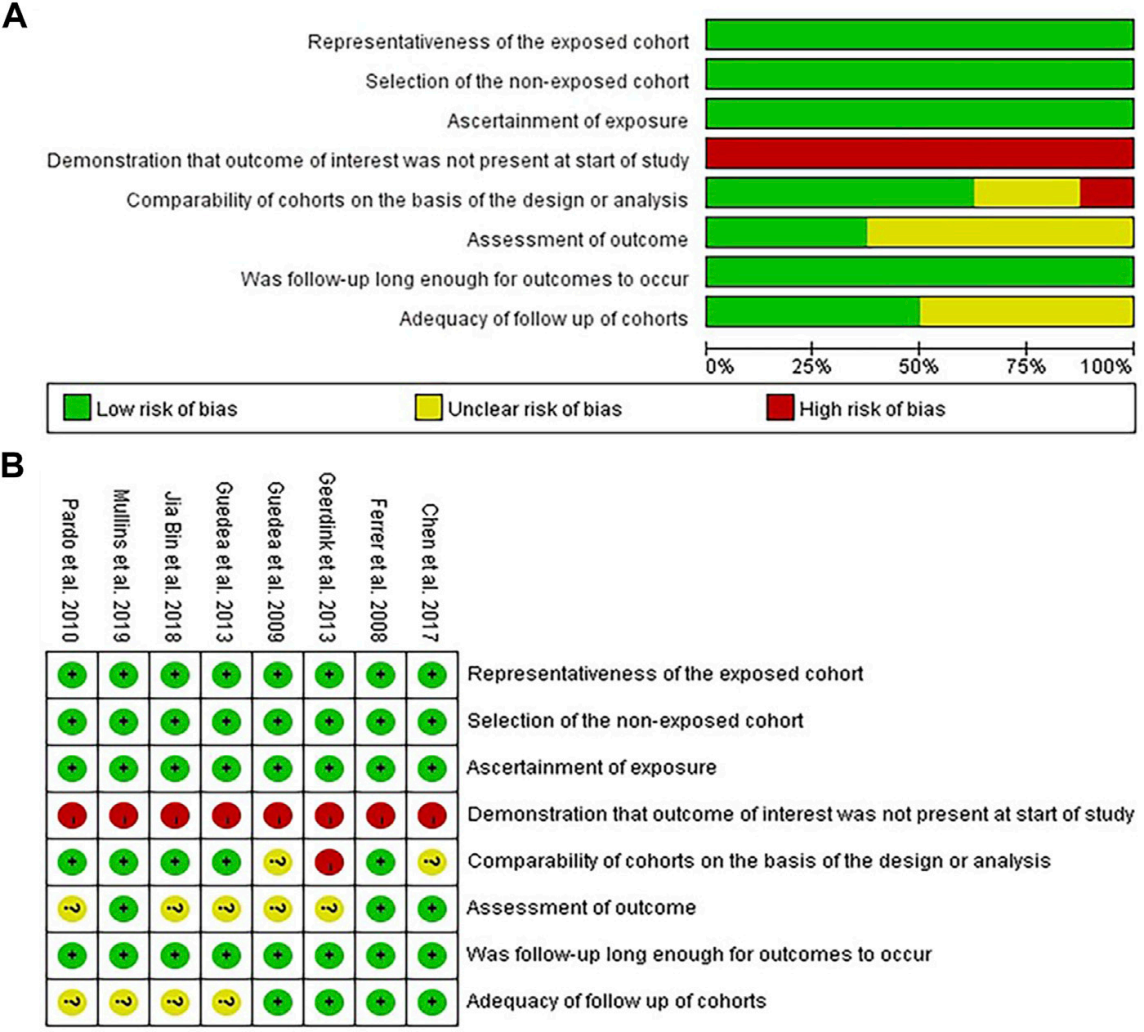
**(A)** Risk of bias graph. **(B)** Risk of bias summary.

The participants included in the 8 articles met the diagnostic criteria for localized PCa and had clear recorded medical histories regarding the treatment. In addition, the follow-up plan was defined appropriately before the evaluation of the sexual function and implemented as scheduled. Three studies (Ferrer et al., 2008; Chen et al., 2017; Mullins et al., 2019) independently evaluated the results using a blind method; four clinical trials (Ferrer et al., 2008; Guedea et al., 2009; van Tol-Geerdink et al., 2013; Chen et al., 2017) specified the missing rate at the beginning or described the lost visits after the trial; and five studies (Ferrer et al., 2008; Pardo et al., 2010; Ferrer et al., 2013; Jia and Zhang, 2018; Mullins et al., 2019) performed stratified or covariate analysis to control for confounding bias, as shown in Figures 2A,B, where “green” represents low risk, “yellow” represents unknown risk, and “red” represents high risk.

### 3.4 Meta-Analysis

#### 3.4.1 Overall Analysis of the Sexual Function Scores

**FIGURE 3:**
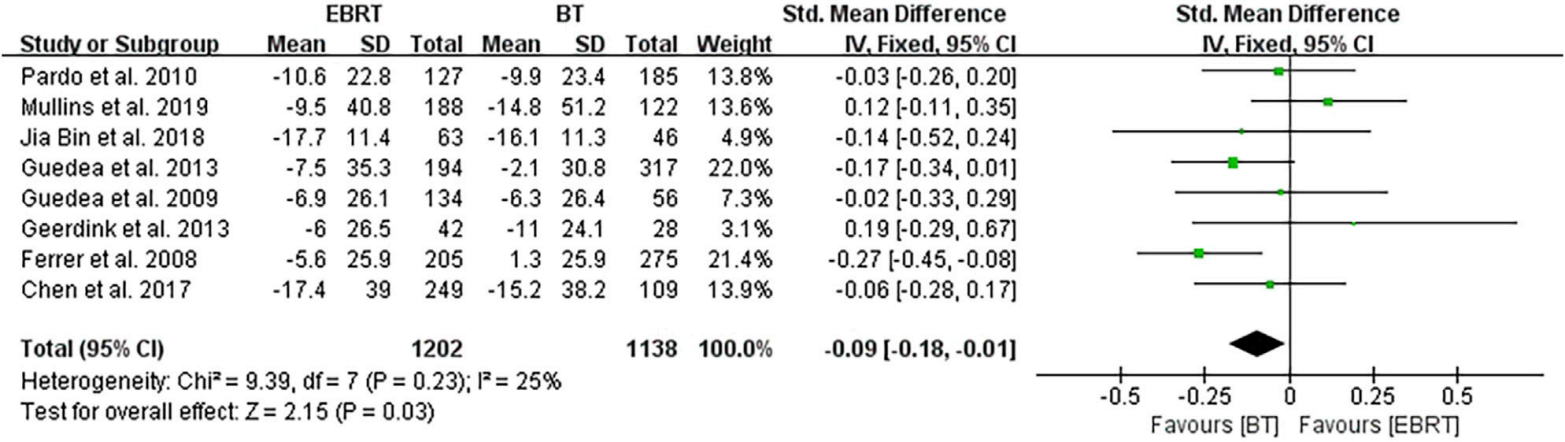
Meta-analysis of two radiotherapy modalities on sexual function scores.

Among the eight studies, five (Ferrer et al., 2008; Guedea et al., 2009; Pardo et al., 2010; Ferrer et al., 2013; van Tol-Geerdink et al., 2013) used the EPIC scale, and three (Chen et al., 2017; Jia and Zhang, 2018; Mullins et al., 2019) used the PCSI scale. Knowing that a higher score in the EPIC scale suggests a better sexual function, the mean PCSI score was dealt with a minus in the forest plot to align the direction of all scales as shown in Figure 3. The overall analysis was performed with the endpoint of each trial as a node. Heterogeneity was acceptable (I^2^ = 25%), so the fixed-effects model was used. The test for overall effect showed that Z = 2.15, SMD = −0.09, 95% CI: −0.18 to −0.01, with the merged interval located to the left of the invalid line and the difference was statistically significant (*p* = 0.03), illustrating that BT had less impact on the sexual function than EBRT in patients with localized PCa.

#### 3.4.2 Subgroup Analysis

**FIGURE 4:**
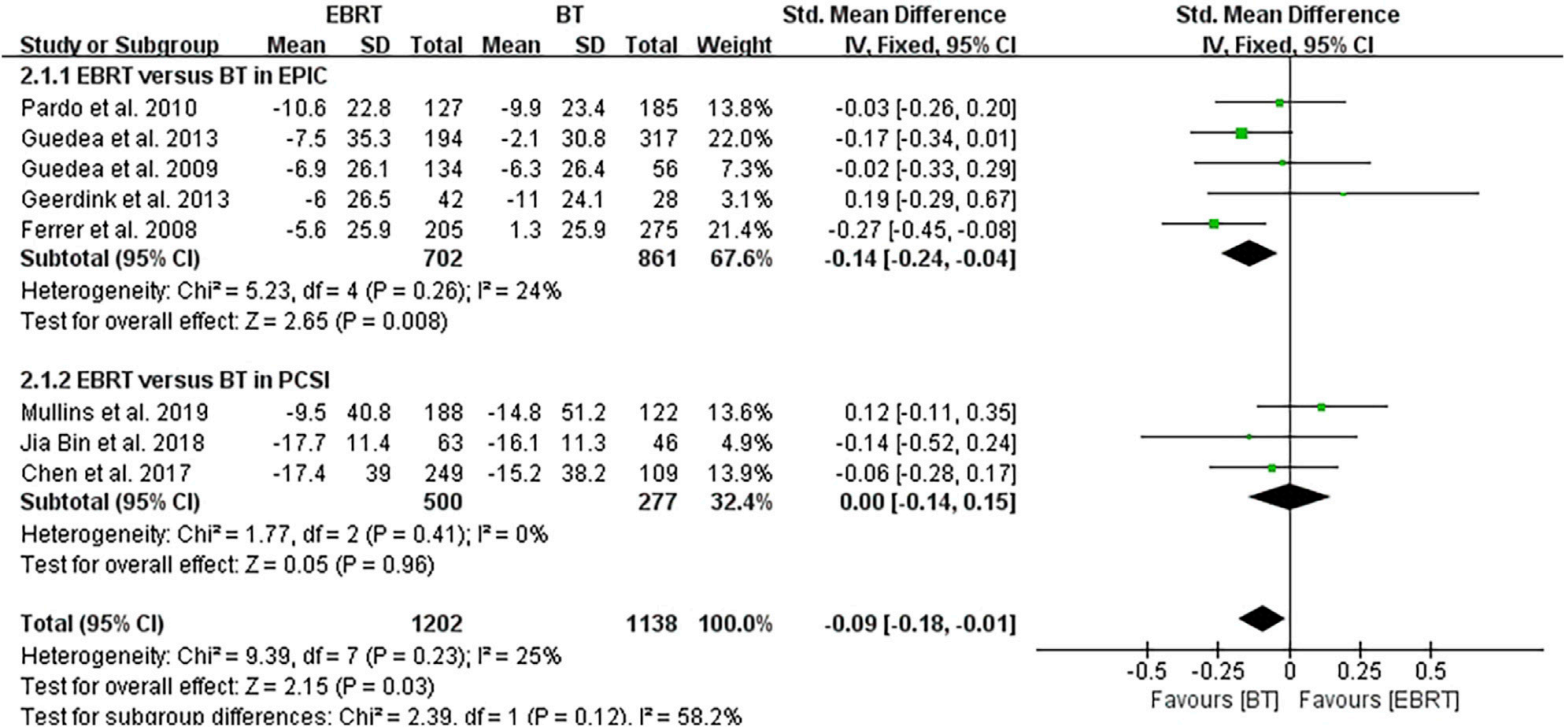
Subgroup analysis of different scales.

Subgroup analysis was performed according to the two scales shown in Figure 4, also using the follow-up endpoint of each study as a node. In five articles using the EPIC scale (Ferrer et al., 2008; Guedea et al., 2009; van Tol-Geerdink et al., 2013; Ferrer et al., 2013; Pardo et al., 2010), heterogeneity was not obvious (I^2^ = 24%), using the fixed-effects model. The test for overall effect indicated that Z = 2.65, SMD = −0.14, 95% CI: −0.24 to −0.04. The combined interval fell to the left of the invalid line (*p* = 0.008), demonstrating that the sexual function of localized PCa was less affected by BT than EBRT by the EPIC scale. In the three articles using the PCSI scale (Chen et al., 2017; Jia and Zhang, 2018; Mullins et al., 2019), no heterogeneity was observed (I^2^ = 0%), using the fixed-effects model. The test for overall effect showed that Z = 0.05, SMD = 0.00, 95% CI: −0.14 to 0.15, the merged interval crossed the invalid line, and there was no significant difference (*p* = 0.96).

#### 3.4.3 Effect of Dose on Sexual Function Evaluation in Brachytherapy/External Beam Radiotherapy

**FIGURE 5:**
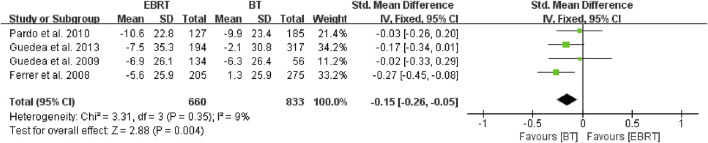
Meta-analysis of sexual function scores with the same dose of BT/EBRT. BT, brachytherapy; EBRT, external beam radiotherapy.

Given that the radiation dose may have some impact on the study, we conducted an in-depth analysis. After careful review, we found that two studies (Chen et al., 2017; Mullins et al., 2019) did not mention radiation dose or cycle, and the remaining six studies (Ferrer et al., 2008; Guedea et al., 2009; Pardo et al., 2010; Ferrer et al., 2013; van Tol-Geerdink et al., 2013; Jia and Zhang, 2018) differed mainly in doses of EBRT (four (Ferrer et al., 2008; Guedea et al., 2009; Pardo et al., 2010; Ferrer et al., 2013) with 74 Gy, one (Jia and Zhang, 2018) with 76–80 Gy, and one (van Tol-Geerdink et al., 2013) with 78 Gy. The doses of BT within the six trials were basically consistent, with the particle being ^125^I and the prescription dose being 144 Gy to the reference isodose (100%) according to the TG-T43 (Bice Jr et al., 1998). So four studies (Ferrer et al., 2008; Guedea et al., 2009; Pardo et al., 2010; Ferrer et al., 2013) were selected for the analysis, including 660 patients receiving EBRT and 833 patients receiving BT (Figure 5). The fixed-effects model was chosen because of heterogeneity (I^2^ = 9%). The test for overall effect showed that Z = 2.88, SMD = −0.15, 95% CI: −0.26 to −0.05, with the combined interval located to the left of the invalid line, with a significant difference (*p* = 0.004).

#### 3.4.4 Comparison of Sexual Function Evaluation at Different Follow-Up Periods

**FIGURE 6:**
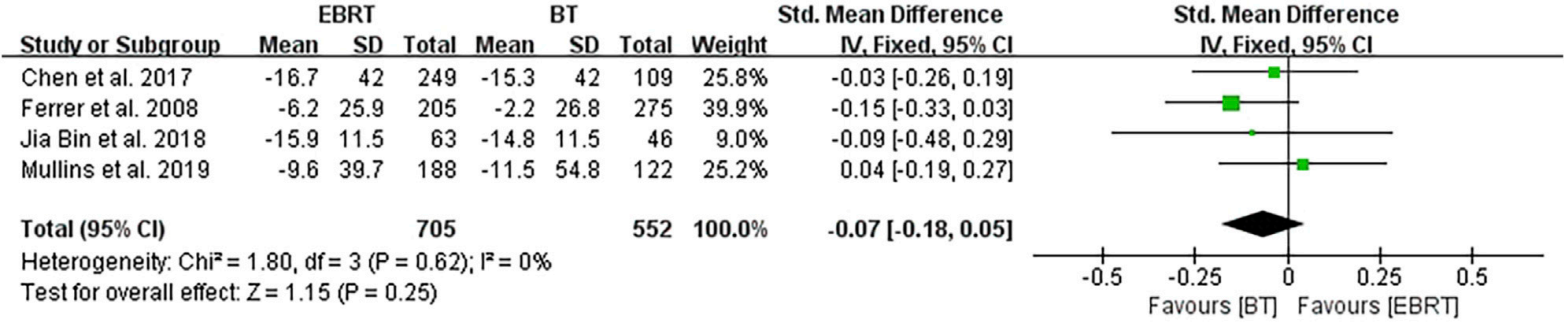
Meta-analysis of sexual function scores at 3 months after radiotherapy.

Four studies (Ferrer et al., 2008; Chen et al., 2017; Jia and Zhang, 2018; Mullins et al., 2019) carried out analysis at 3 months after radiotherapy, involving 705 patients receiving EBRT and 552 patients receiving BT (Figure 6). No heterogeneity was present (I^2^ = 0%), using the fixed-effects model. The test for overall effect suggested that Z = 1.15, SMD = −0.07, 95% CI: −0.18 to 0.05, with the merged interval crossing the invalid line, and the difference was not statistically significant (*p* = 0.25).

**FIGURE 7:**
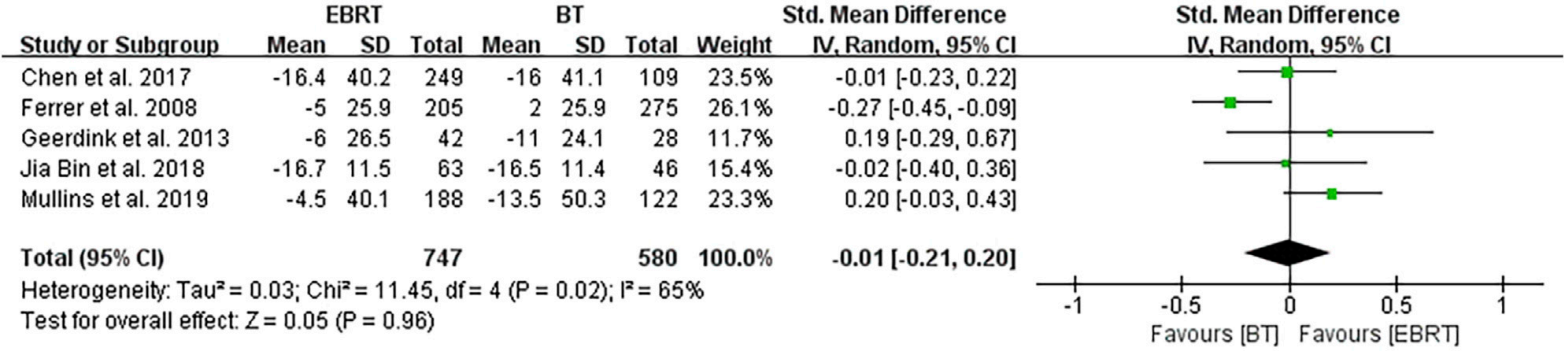
Meta-analysis of sexual function scores at 12 months after radiotherapy.

Five clinical trials (Ferrer et al., 2008; van Tol-Geerdink et al., 2013; Chen et al., 2017; Jia and Zhang, 2018; Mullins et al., 2019) performed the analysis at 12 months after treatment, involving 747 patients receiving EBRT and 580 patients receiving BT (Figure 7). Obvious heterogeneity was observed (I^2^ = 65%), and the random-effects model was applicable. The test for overall effect showed that Z = 0.05, SMD = −0.01, 95% CI: −0.21 to 0.20, with the combined interval crossing the invalid line, and with no statistical difference (*p* = 0.96).

**FIGURE 8:**
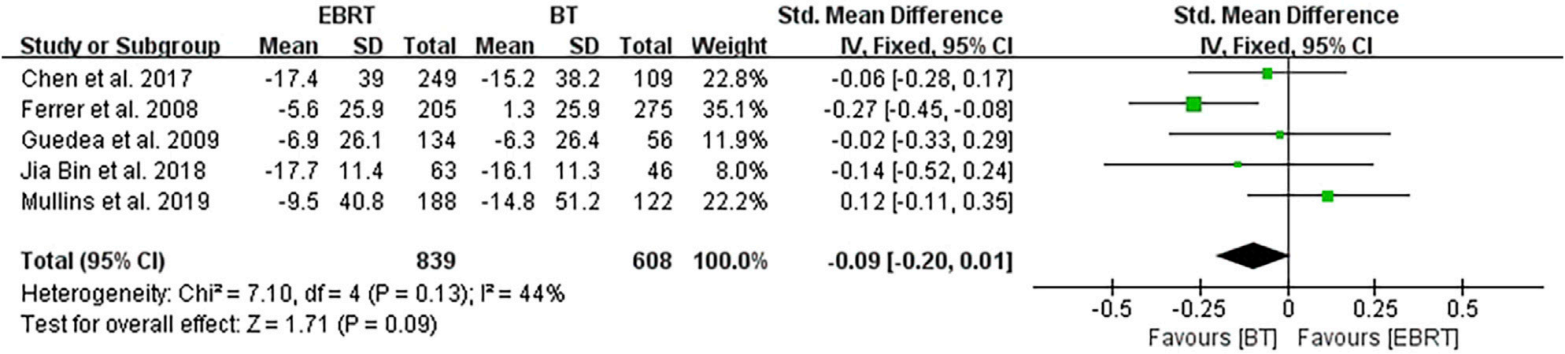
Meta-analysis of sexual function scores at 24 months after radiotherapy.

Five studies (Ferrer et al., 2008; Guedea et al., 2009; Chen et al., 2017; Jia and Zhang, 2018; Mullins et al., 2019) conducted analyses at 24 months after radiotherapy, involving 839 patients receiving EBRT and 608 patients receiving BT (Figure 8). Heterogeneity was evaluated (I^2^ = 44%), using the fixed-effects model. The test for overall effect showed that Z = 1.71, SMD = −0.09, 95% CI: −0.20 to 0.01, with the merged interval across the invalid line, and the difference was not statistically significant (*p* = 0.09).

#### 3.4.5 Sensitivity Analysis

With the use of the RevMan software, the 8 clinical trials were eliminated one by one in sequence for sensitivity analysis. Change in the total combined effect was observed to determine whether the results of the meta-analysis were stable. It was found that the SMD of the combined effect value after exclusion of a single article fluctuated between −0.04 and −0.12, which was basically consistent with the combined total effect value, indicating that the research results were stable.

## 4 Discussion

### 4.1 Background

With the innovation of modern technology, the application of radiotherapy as one of the radical therapies for localized PCa has become increasingly mature. A prospective randomized controlled trial reported that there was no significant difference in cancer-specific survival (CSS) and 10-year OS between radiotherapy and radical surgery for localized PCa (Wallis et al., 2016). EBRT has evolved from traditional rotating irradiation and four-field box irradiation to intensity-modulated radiotherapy (IMRT), stereotactic radiotherapy (SBRT), and three-dimensional conformal radiotherapy (3D-CRT), which increases the dose to the target area but reduces the dose to surrounding normal tissues, thereby substantially reducing the occurrence of complications. BT is the implantation of radioactive particles into the human tissue for the purpose of radiation therapy, including temporary seed implantation and permanent seed implantation. The dose distribution around the radioactive particles is inversely proportional to the square of the distance from the radioactive source, ensuring a high dose to the local lesion tissue and a low dose to the surrounding normal tissue, thus reducing the damage to the surrounding normal tissue, which, together with the implantation of seeds to reduce the scope of surgical anatomy, provides further assurance of reducing impairment to the sexual function (de la Puente and Azab, 2014). Current evidence-based medicine indicates that BT alone is superior to EBRT alone in terms of efficacy and safety for the treatment of localized PCa (Kee et al., 2018; Li et al., 2018), but there is no conclusive evidence to define the difference in the effect of BT versus EBRT on the sexual function. The aim of the present study was to provide a basis for decision-making in the choice of clinical treatment.

### 4.2 Main Findings of the Present Meta-Analysis

After the integrated analysis of the eight articles included in this study, we found that the sexual function was less affected in patients with localized PCa who received BT as compared with that in patients who received EBRT in terms of the integrated scores of EPIC and PCSI. Although the overall heterogeneity was small (I^2^ = 25%), subgroup analysis was carried out to make the study more convincing, because two different scales were adopted in the different studies included. The results showed less intragroup heterogeneity (I^2^ = 24%) in the five studies using the EPIC scale (Ferrer et al., 2008; Guedea et al., 2009; van Tol-Geerdink et al., 2013; Ferrer et al., 2013; Pardo et al., 2010), and there was still a statistically significant difference between BT and EBRT. In contrast, analysis of the three studies using the PCSI scale (Chen et al., 2017; Jia and Zhang, 2018; Mullins et al., 2019) showed no intragroup heterogeneity (I^2^ = 0%), but there was no significant difference in sexual function scores, and with higher heterogeneity between the two subgroups (I^2^ = 58.2%). The result may be due to the smaller number of both studies using the PCSI scale and cases on the one hand; and on the other hand, it may be that the studies using the PCSI scale had a shorter mean follow-up period. Considering that the difference in radiation dose may have a certain influence on the results, we analyzed four studies (Ferrer et al., 2008; Guedea et al., 2009; Pardo et al., 2010; Ferrer et al., 2013) with the same radiation dose. The results revealed less intragroup heterogeneity (I^2^ = 9%), and the difference between BT and EBRT was statistically significant (*p* = 0.004). This also greatly increased the persuasiveness of the article.

In view of the difference in the length of follow-up periods, we further carried out a stratified analysis and found that there was no statistically significant difference in the sexual function scores between the two methods at 3, 12, and 24 months after treatment. The reason may be that fewer studies were included in the stratified analysis, and most included studies used the PCSI scale. For example, four studies (Ferrer et al., 2008; Chen et al., 2017; Jia and Zhang, 2018; Mullins et al., 2019) were included in the 3-month analysis, of which three studies used the PCSI scale; five studies (Ferrer et al., 2008; van Tol-Geerdink et al., 2013; Chen et al., 2017; Jia and Zhang, 2018; Mullins et al., 2019) were included in the 12-month analysis, including three studies using the PCSI scale; and five studies (Ferrer et al., 2008; Guedea et al., 2009; Chen et al., 2017; Jia and Zhang, 2018; Mullins et al., 2019) were included in the 24-month analysis, of which three studies used the PCSI scale. In addition, the follow-up duration may not be long enough, and therefore longer-term follow-up studies are required to verify our findings and conclusions.

### 4.3 Value and Significance

Most of the included articles were prospective cohort studies in the previous 10 years, with a large total number of cases. Although these studies used two different scales to evaluate the sexual function convincing, data analysis showed that there was low heterogeneity and the publication bias was within the acceptable limits, and sensitivity analysis showed that the results were stable, indicating that the conclusions of the present study are reliable and highly convincing.

Patients with PCa not only have high expectations for the therapeutic effect but are very much concerned about the changes in their quality of life. This study confirms that BT, to some extent, has a better therapeutic effect on the sexual function in patients than EBRT and therefore may provide a basis for the choice of clinical treatment.

### 4.4 Limitations

First, this is an observational study, and the results obtained may be affected by various confounding factors. The lack of further grouping of tumors may also increase the study bias. Second, as the included articles were prospective cohort studies without randomized control trials, and the strength of the argument needs to be improved. Third, the original data of some studies were extracted by WebPlotDigitizer or calculated by related formulas and may produce errors in the results. Finally, this study only compared the effects of BT alone versus EBRT alone and did not include combination therapy (BT+EBRT) for comparison. Further in-depth research is required to verify our findings and conclusion.
